# Single-Sided Deafness and Hearing Rehabilitation Modalities: Contralateral Routing of Signal Devices, Bone Conduction Devices, and Cochlear Implants

**DOI:** 10.3390/brainsci14010099

**Published:** 2024-01-20

**Authors:** Alessandra Pantaleo, Alessandra Murri, Giada Cavallaro, Vito Pontillo, Debora Auricchio, Nicola Quaranta

**Affiliations:** 1 Otolaryngology Unit, Department of BMS, Neuroscience and Sensory Organs, University of Bari, 70121 Bari, Italy; alessandrapantaleo18@gmail.com (A.P.); alessandramurri20@gmail.com (A.M.); pontillovito@gmail.com (V.P.); debora.auri@gmail.com (D.A.); 2 Otolaryngology Unit, Madonna delle Grazie Hospital, 75100 Matera, Italy; giadacavallaro@live.it

**Keywords:** single-sided deafness, cochlear implants, contralateral routing of signal devices, bone conduction devices

## Abstract

Single sided deafness (SSD) is characterized by significant sensorineural hearing loss, severe or profound, in only one ear. SSD adversely affects various aspects of auditory perception, including causing impairment in sound localization, difficulties with speech comprehension in noisy environments, and decreased spatial awareness, resulting in a significant decline in overall quality of life (QoL). Several treatment options are available for SSD, including cochlear implants (CI), contralateral routing of signal (CROS), and bone conduction devices (BCD). The lack of consensus on outcome domains and measurement tools complicates treatment comparisons and decision-making. This narrative overview aims to summarize the treatment options available for SSD in adult and pediatric populations, discussing their respective advantages and disadvantages. Rerouting devices (CROS and BCD) attenuate the effects of head shadow and improve sound awareness and signal-to-noise ratio in the affected ear; however, they cannot restore binaural hearing. CROS devices, being non-implantable, are the least invasive option. Cochlear implantation is the only strategy that can restore binaural hearing, delivering significant improvements in speech perception, spatial localization, tinnitus control, and overall QoL. Comprehensive preoperative counseling, including a discussion of alternative technologies, implications of no treatment, expectations, and auditory training, is critical to optimizing therapeutic outcomes.

## 1. Introduction

Single-sided deafness (SSD) is defined as severe to profound sensorineural hearing loss in one ear and normal or near-normal hearing in the other. However, there is currently no consensus on standardized audiological criteria for defining SSD. The audiological classification criteria for SSD candidate groups, as outlined by Van de Heyning et al. [1] and Ramos Macías et al. [2], are summarized in Table 1.

The lack of a consistent definition and confusion over nomenclature has made it challenging to clearly define the prevalence of SSD. According to Kay-Rivest et al. [3], the prevalence of SSD in the United States is estimated to be between 0.11% and 0.14% and is higher in individuals 60 to 79 years of age. The prevalence of SSD in children is low. Dewyer et al. [4] found an estimated prevalence of 0.36% in a recent retrospective study of 52,878 individuals undergoing behavioral threshold testing at a single tertiary referral center. Before universal neonatal hearing screening programs, the diagnosis of unilateral hearing loss in children was often delayed as compared to those with bilateral sensorineural hearing loss [5]. According to a 1960 study by Everberg et al. [6], 52% of cases of unilateral deafness were not identified until after the first year of school, with an average age of 6 years at the time of symptom recognition. Nowadays, the advent of universal newborn hearing screening programs has significantly lowered the average age of diagnosis [7,8].

SSD may result from different etiopathological mechanisms and may vary in presentation between pediatric and adult populations. According to Usami et al. [9], in children, early-onset congenital SSD is often secondary to cochlear nerve deficiency (CND), CMV and mumps infection, and inner ear abnormalities (e.g., incomplete type I partition or common cavity). Only a small number of cases of early-onset SSD are attributable to genetic causes (e.g., Waardenburg syndrome), which are instead more frequently associated with bilateral sensorineural hearing loss [4]. Children with unilateral hearing loss (UHL) may progress to bilateral deafness; according to Fitzpatrick et al. [10], of 537 children diagnosed with unilateral deafness at birth, 42.2% experienced hearing deterioration over the years, and in 19% of cases it eventually developed into bilateral deafness. The most frequent cause of SSD in adults is sudden hearing loss stemming from different etiologies (e.g., Mèniére disease [11], idiopathic [12,13], and autoimmune diseases [14,15]); SSD may also present as progressive hearing loss, such as in chronic otitis media with and without cholesteatoma [9], or vestibular schwannoma [16,17].

SSD is often associated with various symptoms that can significantly impair a person in daily life. Among these, tinnitus–characterized by the perception of noise without an external source–is a common problem; in fact, between 54 and 84% of adults with SSD experience debilitating tinnitus [18,19]. Along with tinnitus, patients with SSD may also experience hyperacusia [20], aural fullness, and changes in the vestibular system, especially in cases of cochleo-vestibular impairment such as in Mèniére disease [11,21].

Despite being undertreated in the past due to the misconception that the unaffected ear was sufficient for general speech development in early prelingual cases and adequate for acceptable hearing function in adults, it is now widely recognized that the management of unilateral hearing loss is crucial for both children and adults. SSD can have significant negative effects on an individual’s ability to localize sounds [22,23,24], understand speech in noisy environments [25,26,27], and maintain spatial awareness, leading to a decreased quality of life (QoL) and increased social isolation [28]. In children, SSD-associated lack of binaural information and reduced spatial abilities [29], especially in complex sound environments (e.g., classrooms, schools, and playgrounds), can result in impaired linguistic and academic performance [30,31], cognition [32], and QoL [33].

Cochlear implants (CIs), contralateral routing of signal (CROS) devices, and bone conduction devices (BCD) are possible treatment options for SSD. Although several treatment options are available for the management of SSD, the debate regarding the most effective approach is still ongoing. A major challenge in determining the most appropriate therapeutic intervention for unilateral deafness (SSD) is the lack of unanimous consensus on outcome domains and measurement tools. An early attempt to establish a consensus among cochlear implant (CI) professionals for minimum outcome measures was made by Van de Heyning et al. [1]. More recently, the CROSSSD (Core Rehabilitation Outcome Set for Single Sided Deafness) initiative [34] reached an international consensus by incorporating the perspectives of users and health professionals. This initiative identified three core outcome domains: spatial orientation, group conversations in noisy social situations, and impact on social situations. However, the tools to measure these outcomes have yet to be determined. Adopting a common protocol will facilitate high-quality studies in the future and allow easier comparison of results.

This narrative overview aims to summarize the scientific evidence to date on different treatment options for SSD in adult and pediatric populations, hearing outcomes, and their impact on QoL.

## 2. Consequences of SSD in Adults

Binaural cues are essential for the localization and perception of target sounds, especially in the presence of noise. Depending on the location of the sound source, in fact, since the two ears are physically separated from the head, the signals reaching each ear may differ in time of arrival (interaural time difference—ITD) or intensity (interaural level difference—ILD). Other advantages of binaural listening include the “head shadow effect” (which causes listeners to focus on the ear with a better sound-to-noise ratio), “binaural summation” (a special case of binaural redundancy), and the “squelch effect” (which allows the brain to suppress competing noise for better perception of speech in noise) [35].

In individuals with normal hearing (NH), unilateral auditory stimulation evokes predominantly contralateral activation in the auditory cortex (contralateral dominance) [36,37]. Unilateral hearing loss with disruption of binaural inputs results in brain reorganization with a weakening in the representation of the deprived ear and a strengthening in the representation of the intact ear (“auditory preference syndrome”) [38,39]. Brain reorganization is detectable 5 weeks after the onset of SSD in adults. Functional magnetic resonance imaging (fMRI) studies have shown that reorganization stabilizes after 1 year [40] and that the dominance shift also affects the nonprimary auditory cortex (NPAC) [41].

As a consequence of the loss of binaural advantages, SSD patients experience greater difficulty in speech perception in noise and sound localization, with several functional limitations. These include safety risks such as not hearing a vehicle or bicycle approaching from the deaf side, as well as an elevated cognitive load necessary for processing auditory information.

SSD has been associated with increased levels of anxiety, difficulty in communication in the presence of background noise, and decreased self-esteem [28,42]. Difficulty in communication with multiple stakeholders leads SSD patients to withdraw from social situations, impacting personal and professional relationships. The impact of SSD also extends to general well-being. Studies have shown that people with unilateral hearing loss are more likely to report poor health, dissatisfaction, and loneliness than those with normal hearing. Furthermore, even with the use of hearing aids, patients with SSD often experience a decline in health-related quality of life [26,43].

## 3. Rerouting Solutions

### 3.1. Controlateral Routing of Signal (CROS)

Conventional approaches to hearing rehabilitation designed for SSD typically involve rerouting the auditory signal from the affected ear to the unaffected or better-functioning ear to facilitate further processing. Contralateral routing of signal (CROS) devices provide a non-surgical approach and represent the least invasive solution currently available. CROS hearing aids consist of a microphone and transmitter in a hearing aid worn on the impaired ear, which transmits sound to the functioning ear either via a wire or wirelessly [44]. In cases of asymmetric hearing loss (sensorineural, conductive, or mixed), the aid on the better hearing ear can also provide amplification in addition to the CROS input, creating a configuration known as bilateral contralateral routing of signals (BiCROS). BiCROS is typically recommended for individuals with mild to moderate hearing loss in the better hearing ear [45].

Recently, a new technology has been proposed for individuals with SSD who experience bothersome tinnitus in the poorer ear, along with difficulties in understanding speech in noise and sound localization. The new device combines the ability to reroute the sound from the poorer ear to the good ear (CROS system) while still providing bilateral stimulation with conventional amplification (StereoBiCros). This strategy seems to reduce tinnitus handicap and loudness for individuals with AHL/SSD [46]. The underlying mechanism of the positive effect of StereoBiCros on tinnitus is unclear; it is likely due to the acoustic masking of tinnitus produced by the acoustic stimulation of the poorer ear. Furthermore, this stimulation could reverse tinnitus-related central plasticity [47].

Evidence suggests that CROS devices are successful in reducing the negative effects of acoustic head-shadow and enhancing awareness of sound and the signal-to-noise ratio (SNR) when sounds are directed toward the affected ear [24,48,49,50]. The design, particularly the small and unobtrusive housing of current wireless CROS and BiCROS hearing aids, is certainly an added advantage, influencing the acceptance of this solution. These devices are also easy to manage, especially in patients with SSD, where there is no contralateral hearing loss, and thus, no sophisticated programming or fitting strategies are required [49]. However, CROS solutions do not restore binaural hearing and cannot improve tasks that require binaural cues, such as sound localization abilities in the horizontal plane [51,52,53]. Furthermore, in listening situations where the signal of interest is in the better ear and noise is transferred through the CROS microphone from the affected side, there is a significant reduction in speech comprehension [49].

### 3.2. Bone Conduction Devices (BCD): Surgically Implanted Devices

Bone conduction devices (BCD) are rerouting devices that transmit signals from the ear with SSD to the better ear via bone conduction, bypassing the air conduction pathway [54]. Since the late 1970s, when the first bone-anchored hearing aids (BAHA) were implanted, many different devices, both implantable and non-implantable, have been introduced.

Surgically implanted BCD transform sound waves into mechanical vibrations through direct contact with the skull, facilitating transmission to the inner ear. Although they share essential components, BCD can be divided into two distinct types: percutaneous and transcutaneous devices. Transcutaneous devices can be further distinguished as passive or active types. In percutaneous devices (e.g., Oticon Ponto System [55] and Cochlear™ Baha^®^ Connect System [56]), sound detected by the external processor is transformed into vibrations transferred through a percutaneous osseointegrated pin or abutment in the skull. This direct connection allows efficient signal transmission at all frequencies, without skin or soft tissue impedance. However, complications, including skin reaction, granulation tissue formation, keloids, and soft tissue infection are not uncommon [57]. In adults a one-stage surgery is generally performed; conversely, for children or individuals with compromised bone mineralization, such as those with post-radiation bone issues where osseointegration failure is prevalent, a two-stage procedure should be considered. The first stage involves implant placement to facilitate osseointegration, followed by the second stage to install a stump that extends through the skin.

Passive transcutaneous devices (e.g., Cochlear™ Baha^®^ Attract [56] and Medtronic Alpha 2 MPO ePlus™ [58]) consist of an implanted part, similar to percutaneous devices, and an external part held in place magnetically, which transmits vibrations transcutaneously to the implanted device, avoiding the need for a skin penetrating stump. The major advantage of these devices is their lower frequency of skin complications. However, there is some sound attenuation caused by soft tissue, reaching up to 25 dB at 6000–8000 Hz [59] when compared with percutaneous implants. Additionally, they may cause discomfort due to the magnetic force required to secure the processor [60]. Symptoms can be alleviated by reducing the pressure of the magnet and limiting use of the device. However, excessive pressure can lead to skin necrosis if it exceeds capillary pressure [61].

To maximize the benefits of percutaneous and transcutaneous devices, transcutaneous active BCD such as the Bonebridge™ [62] and the Osia^®^ System [63] have been developed. Active transcutaneous devices consist of an external audio processor (containing a microphone, processor, and battery) and an internal system that houses the magnet, coil, and actuator, also known as a floating mass bone conduction transducer (BC-FMT). The FMT is attached to the skull using cortical fixation screws that do not require osseointegration [64]. Sound waves are electrically transmitted from the external device to the internal device using technology similar to that of cochlear implants. Since it is the internal device that is responsible for generating mechanical forces against the skull, skin attenuation is minimized and magnet power can be greatly reduced [60]. The device is commonly implanted in the pre-sigmoid mastoid bone. Alternative positions, such as the retrosigmoid or through the middle fossa, may be necessary in cases where the sigmoid sinus is too anterior, the dura mater in the middle cranial fossa is too low, or if the patient has previously undergone mastoidectomy [65].

BCD can alleviate the shadow effect and improve sound awareness on the affected side [44,66]; however, they cannot restore binaural hearing and evidence suggests that these devices do not improve sound localization ability [44,66,67]. Several studies report the reduction of tinnitus by BCD [67,68,69,70]. Indeed, it is believed that stimulation of the contralateral auditory pathway may play an important role in suppressing experienced tinnitus [71]. Regarding the effects of BCD on QoL, a recent systematic review and meta-analysis found that BCD are associated with significant improvements in hearing-related QoL as measured by the APHAB (Abbreviated Profile of Hearing Aid Benefit) https://harlmemphis.org/abbreviated-profile-of-hearing-aid-benefit-aphab/ (accessed on 10 January 2024) and SSQ (Speech, Spatial, and Qualities of Hearing Scale) scores in adult patients, while no difference was found in generic measures of QoL as measured by the HUI-3 (Health Utilities Index-3) [72].

Discomfort with the device, concerns about sound quality, and subjective auditory impairment are among the main reasons for rejecting CROS and BCD [73,74]. Reluctance to use redirecting devices is also linked to changes in self-perception, aesthetic concerns, and the presence of negative stereotypes. Anxiety about surgery is also a common reason for rejecting BCD [74].

### 3.3. Bone Conduction Devices (BCD): Extrinsic Devices

Non-implantable bone conduction hearing devices are available for patients who are not candidates for surgery, those uninterested in surgery, or as a pre-implantation simulator for individuals considering a surgically implanted device. Extrinsic devices transmit sound vibrations through intact skin, to which they are attached through means of bands, soft bands, adhesives, and glasses. However, these devices are less effective than osseointegrated systems because they experience signal attenuation through the skin and soft tissues, especially at high frequencies, and their viable duration of use may be limited because of the discomfort caused by the force required to secure them in place [60,75].

Non-implantable bone conduction devices include the adhesive bone conduction device (ADHEAR), developed by MED-EL, designed for patients with unilateral deafness or conductive hearing loss with a bone conduction PTA better than or equal to 25 dB HL [76]. ADHEAR is a bone-conduction hearing solution known for its distinctive adhesive attachment method. Specifically, the device is affixed to the skin over the mastoid bone using a specialized adhesive, ensuring secure placement without causing discomfort or pressure-related issues.

In a study by Mertens et al. [77], 17 SSD patients participated in a prospective randomized crossover study comparing an adhesive hearing system with a CROS hearing aid. Group A started with the adhesive device, and Group B with the control device, followed by a crossover test after 2 weeks. The results showed that 70% of SSD-affected participants found the adhesive system partially useful or better, with satisfaction levels similar to those using the control device according to the Audio Processor Satisfaction Questionnaire (APSQ). While sound localization improved with the adhesive system, there was no significant improvement in speech perception in noisy environments.

Another non-implantable bone conduction device is SoundBite [78], developed by Sonitus Technologies. It consists of a behind-the-ear microphone (BTE) placed in the damaged ear, capturing sound that is then processed by digital audio equipment inside the BTE. Additionally, there is a removable device inside the mouth (ITM), specially designed to fit comfortably in the upper back teeth, which receives the signals processed by the BTE device, converting them into vibrations, which stimulate the cochlea, allowing the user to perceive the sound. According to a study conducted by Lou et al. [79] on nine patients with SSD, SoundBite has been shown to lead to improved speech recognition and overall quality of life in both quiet and noisy environments.

## 4. Cochlear Implants (CI)

A cochlear implant (CI) is a surgically implanted electronic device that contains an array of electrodes which is placed into the cochlea and stimulates the cochlear nerve, bypassing the injured parts of the inner ear. Initially suggested as a treatment for severe tinnitus in adults with single-sided deafness (SSD), cochlear implant provision and rehabilitation has now become the clinical standard for SSD. In 2019, the FDA expanded the indications for cochlear implantation to include individuals aged 5 years and older with profound sensorineural hearing loss in the compromised ear (PTA: 5, 1, 2, and 4 kHz of >80 dB HL) and normal hearing (NH) in the contralateral ear (PTA: 5, 1, 2, and 4 kHz ≤30 dB HL) [80,81].

The outcomes of cochlear implantation (CI) are closely related to the integrity of the cochlea and cochlear nerve (CN) [82,83]; therefore, CI is traditionally contraindicated in cases of cochlear nerve deficiency (CND), such as CN aplasia and hypoplasia. CND could be detected by computed tomography (CT) measurement of the inner auditory canal (IAC) and cochlear nerve bone canal (BCNC) diameters [84]. However, a normal IAC is not a reliable marker of a normally developed cochlear nerve [85]. Hence, especially in children with SSD, given the high prevalence of CND, magnetic resonance imaging (MRI) is recommended over CT alone to confirm the CN condition [86].

A systematic review and meta-analysis conducted by Oh et al. [87] on 50 studies involving 674 adult patients with SSD who underwent ipsilateral cochlear implantation showed statistically significant improvement in all domains of interest. These domains include speech perception, tinnitus reduction, sound localization, and global and disease-specific quality of life (QoL). Similar results were found in previous research by Van Zon et al. [88] and Junior et al. [89], as well as in the results of two tinnitus-specific systematic reviews by Peter et al. [90] and Levy et al. [91].

Karoui et al. [92] demonstrated that restoration of binaurality results in reversal of the abnormal cortical lateralization pattern in UHL subjects, resulting in improved spatial hearing.

Continued improvements in localization have been demonstrated in cochlear implant users with SSD after long-term use of the cochlear implant. According to Thompson et al. [93], adult cochlear implant wearers with SSD showed significant enhancements in their sound localization abilities in the first few weeks after activation, and this improvement was sustained after one year of CI use. Moreover, localization accuracy and consistency continued to improve over the five-year follow-up period after activation.

Advanced age is not a contraindication for cochlear implants (CI), which have risks and individual performance outcomes for patients of an advanced age similar to those observed in younger adults [94].

Several studies have shown a clear negative correlation between duration of deafness (DoD) and CI performance in SSD individuals, due to the effect of prolonged monoaural hearing on the auditory pathways [95]. However, long duration of deafness for adults with SSD should not be the sole contraindication to CI. Rader et al. [96], in their retrospective analysis involving 36 adults with post lingual deafness, found a more favorable result in speech perception 12–36 months post CI activation in patients with a duration of deafness of fewer than 400 months. For those with a longer duration, success is limited, but still possible. Similar findings were demonstrated by Nassiri et al. [97], who observed no difference in speech perception among SSD patients with CI, regardless of whether their deafness had lasted more or less than 10 years.

Studies comparing reorientation technologies with CI in SSD adults revealed that CIs significantly enhance sound localization. Additionally, CI users demonstrate equal or significantly better performance in measures of speech recognition in noise and subjective benefit [98,99].

Auditory training has been shown to be effective in improving the performance of conventional CI users [100,101]. Several studies have supported the importance of intensive auditory training in CI users with SSD. The aim is to foster the perceptual integration of the electrically stimulated ear (i.e., the ear with CI) with the dominant hearing ear, thereby providing subjects with binaural hearing and optimizing results [1,102,103]. Further studies regarding the effectiveness of optimal auditory training methods, and to determine the recommended timing for individuals with SSD using CI, are needed.

## 5. SSD in Pediatric Population

Auditory deprivation resulting from monaural sensory input in children with unilateral deafness (SSD) may have a significant impact on the development of auditory pathways and brain networks associated with higher-order cognitive functions [104,105]. It has been shown that unilateral deafness has implications in language development [31], cognition [32], and quality of life [33], with greater listening-related fatigue [106] in children and difficulties in school learning compared with normal hearing children [107,108].

Children with SSD aged 9 to 14 years have reduced accuracy and efficiency in phonological processing and appear to have impaired executive control function [109]. Confirming this, application of the MRI with diffusion tensor imaging (DTI) technique also shows non-integrity in auditory and associative nonauditory areas during the performance of executive functions in children with SSD compared with normo-hearing children of the same age [110]. Indeed, it has been hypothesized that children with unilateral deafness have different patterns of functional connectivity responsible for auditory and executive functions, which may thus explain behavioral and educational difficulties. Confirmation of this hypothesis comes from resting-state functional connectivity MRI (rs-fcMRI) studies: in the cortical networks supporting executive functions in children with unilateral deafness, there are areas that have adaptive (i.e., strengthened) and maladaptive (i.e., weakened) functional cortical networks, with a lack of predefined suppression in these networks [111]. These findings provide a possible explanation for the educational difficulties experienced by children with unilateral hearing loss. Although studies are few and there is bias in enrollment and etiology, there thus seems to be a direct link between unilateral deafness and cognitive development. Quality of life also appears to be impaired in children and adolescents with SSD in domains related to school performance and social interaction as compared to their normal-hearing counterparts [112].

As with their adult counterparts, several therapeutic strategies including CROS, BCD, and CI have been proposed to address the challenges posed by unilateral deafness, enhancing communication skills, supporting school progress, and improving the overall quality of life of children with SSD.

In children, redirection technologies such as CROS require the ability to handle the device and manage the surrounding environment to avoid transmission of weak signals from the deaf side to the ear with better hearing, as well as adequate ear canal dimensions to accommodate the device and prevent obstruction of the better ear. Bone conduction surgical devices (BCD) are often not available for children under the age of 5 in many jurisdictions or states [113,114].

Data on the audiological benefits of CROS aids and BCD in children with SSD are limited, and moreover, both options fail to provide binaural input. Therefore, they are not generally recommended for children with SSD [115]. In a retrospective cohort study, Chandrasekar et al. [116] found a statistically significant improvement in auditory outcomes, as measured by Children’s Home Inventory for Listening Difficulties (CHILD) questionnaire scores and hearing thresholds for speech in noise, using a BCD compared with no amplification. Similar results were found by Christensen et al. [117,118]. However, in their study, Chandrasekar et al. also observed a low level of adherence to the use of bone conduction devices. Despite improved audiological outcomes and CHILD scores, some patients chose not to adopt amplification. Potential reason for parental rejection seems to be concern about cosmetic appearance and its impact on social acceptance [119].

The only treatment option that can restore bilateral auditory stimulation is cochlear implantation. In 2019, the FDA approved the MED-EL CI in patients with SSD 5 years of age and older. As suggested by Park et al. [115], in children with SSD, the use of these devices at an earlier age could be beneficial, given the importance of neuroplasticity in CI outcomes. Polonenko et al. [120] demonstrated a rapid improvement in cortical reactivity, as measured by electroencephalogram, after a few months of device use in children who received an implant before 3.6 years of age. In contrast, brain reorganization in response to SSD could hinder central binaural integration after cochlear implantation, potentially as early as 2 years after the onset of HL [121,122,123].

Several studies conducted in children with bilateral loss have shown better results in language and speech recognition in children who receive CI early than in those who receive it later [124,125], and a longer duration of deafness has a negative impact on speech recognition [126]. However, these factors should not be considered a limitation to CI in children with unilateral sensorineural hearing loss. Favorable health-related quality of life (HR-QoL) benefits were reported also in children with an older age at implantation and longer durations of deafness [127].

Another factor that may affect the outcomes of CI patients that should be included in CI outcome studies is the time of device use. Indeed, daily device use has been shown to influence CI performance in children with bilateral hearing loss [128,129] and Park et al. [130] observed that in children with SSD and CI, better word recognition was associated with more hours of daily CI use.

Many studies are focused on the evaluation on the benefits of CI in older children. Effectively, assessing the benefits of CI (e.g., in terms of sound localization and perception of speech in noise) in younger children with SSD can be difficult, given limited cooperation and the need for an advanced level of task understanding. The identification of objective measures that do not require the active participation and cooperation of the child is desirable and necessary to better establish the benefits of CI in younger children. In this light, measurement of auditory cortical evoked potentials (CAEPs) to vocal stimuli could serve as a cortical biomarker of audibility and auditory developmental processing efficacy in children with SSD who use a CI, which could prove useful for management [131].

In cases of children with SSD at risk of progressive hearing loss in the better ear (e.g., in cases of CMV), implantation before hearing deterioration in the better ear is recommended to minimize hearing deprivation and improve hearing outcomes. Children with SSD due to bacterial meningitis should be implanted promptly. Cochlear nerve deficiency is a contraindication for cochlear implantation to resolve SSD. Accurate diagnosis of nerve deficit is important, given the high prevalence in children with SSD [115].

In the decision-making path of treatment strategy, it is therefore crucial to carefully consider the duration and etiology of deafness; however, it is also necessary to identify the needs and goals of the family.

## 6. Conclusions

Decision-making for patients with SSD is complex and multifactorial. The lack of unanimous consensus on outcome domains and measurement tools makes it challenging to compare different treatment options.

The main advantages and disadvantages of the various treatment options are summarized in Table 2. Rerouting devices (CROS and BCD) alleviate the head shadow effect and improve sound awareness and signal-to-noise ratio in the affected ear. However, they do not restore binaural hearing. CROS devices, being non-surgically implantable, are the least invasive option. Among BCD, percutaneous BCD often involve skin complications, while passive transcutaneous BCD avoid these problems but may cause discomfort because of the magnetic force required to fix the process. Active transcutaneous BCD solve these drawbacks but require a larger implantation space. Reluctance to adopt redirection devices is also associated with changes in self-perception, aesthetic concerns, and negative stereotypes.

Cochlear implantation (CI) is distinguished by its ability to restore binaural hearing, producing significant improvements in speech perception, spatial localization, tinnitus control, and overall quality of life. However, CI is not suitable for cases of cochlear nerve deficiency (CND), a relatively common cause of congenital SSD. Surgical anxiety contributes to the rejection of BCD and CI.

Appropriate preoperative counseling, including discussions of alternative technologies, implications of treatment avoidance, expectations, and auditory training, is essential to maximize therapeutic benefits.

## Figures and Tables

**Table 1:** Audiological classification criteria for SSD candidate groups.

Authors	Poorer Ear	Better Ear	Interaural Threshold Gap
Van de Heyning et al. [1]	PTA ≥ 70 dB HL	PTA ≤ 30 dB HL	≥40 dB HL
Ramos Macías et al. [2]	Lack of improvement with conventional acoustic aid	≥20 dB HL	NA

PTA: pure tone average; NA: not available.

**Table 2:** Main disadvantages and disadvantages of various treatment options.

Treatment Option	Principles	Advantages	Disadvantages
Contralateral Routing of Signal Devices (CROS)	Rerouting auditory signal from the impaired ear to the better ear	Non-surgically implantable, less invasive Evidence of reduced head shadow effect Evidence of improved sound awareness and signal-to-noise ratio (SNR) when sounds are directed toward the affected ear	Do not restore binaural hearing
Bone Conduction Devices (BCD)	Transmitting signals from the impaired ear to the better ear via bone conduction	Evidence of reduced head shadow effect and improved sound awareness on the affected side. Evidence of tinnitus reduction	Do not restore binaural hearing Invasive, surgically implantable Potential discomfort due to vibrations (active transcutaneous devices) Skin complications (percutaneous devices)
Cochlear Implants (CI)	Surgically implanted device stimulating the cochlear nerve	Restore binaural hearing Evidence of improved speech perception in noise and sound localization Evidence of tinnitus reduction	Invasive, surgically implantable Contraindicated in cases of cochlear nerve deficiency (CND)

## Abbreviations

ADHEAR	Adhesive Bone Conduction Device
AHL	Asymmetric Hearing Loss
APHAB	Abbreviated Profile of Hearing Aid Benefit
BCD	Bone Conduction Devices
BiCROS	Bilateral Contralateral Routing of Signals
cBTE	Behind-The-Ear Microphone
CAEPs	Cortical Evoked Potentials
CI	Cochlear Implant
CMV	Cytomegalovirus
CND	Cochlear Nerve Deficiency
CROS	Controlateral Routing of The Signal
DoD	Duration Of Deafness
FDA	Food And Drug Administration
fMRI	Functional Magnetic Resonance Imaging
HUI-3	Health Utilities-3
ILD	Interaural Level Difference
ITD	Interaural Time Difference
NPAC	Nonprimary Auditory Cortex
PTA	Pure Tone Average
QoL	Quality Of Life
rs-fcMRI	Resting-State Functional Connectivity MRI
SNR	Signal/Noise Ratio
SSD	Single Sided Deafness
SSQ	Speech, Spatial and Qualities Hearing Scale
